# Unique Variants of Avian Coronaviruses from Indigenous Chickens in Kenya

**DOI:** 10.3390/v15020264

**Published:** 2023-01-17

**Authors:** Henry M. Kariithi, Jeremy D. Volkening, Iryna V. Goraichuk, Leonard O. Ateya, Dawn Williams-Coplin, Tim L. Olivier, Yatinder S. Binepal, Claudio L. Afonso, David L. Suarez

**Affiliations:** 1 Exotic and Emerging Avian Viral Diseases Research Unit, Southeast Poultry Research Laboratory, U.S. National Poultry Research Center, USDA-ARS, 934 College Station Rd, Athens, GA 30605, USA; 2 Biotechnology Research Institute, Kenya Agricultural and Livestock Research Organization, Kaptagat Rd, Loresho, Nairobi P.O. Box 57811-00200, Kenya; 3 BASE2BIO, Oshkosh, WI 54904, USA; 4 National Scientific Center Institute of Experimental and Clinical Veterinary Medicine, 83 Pushkinska St., 61023 Kharkiv, Ukraine

**Keywords:** AvCoV, cloacal, gastroenteric, IBV lineage, live bird market, NGS, recombinant, TCoV

## Abstract

The avian gamma-coronavirus infectious bronchitis virus (AvCoV, IBV; *Coronaviridae* family) causes upper respiratory disease associated with severe economic losses in the poultry industry worldwide. Here, we report for the first time in Kenya and the Eastern African region two novel AvCoVs, designated IBV/ck/KE/1920/A374/2017 (A374/17) and AvCoV/ck/KE/1922/A376/2017 (A376/17), inadvertently discovered using random nontargeted next-generation sequencing (NGS) of cloacal swabs collected from indigenous chickens. Despite having genome organization (5′UTR-[Rep1a/1ab-S-3a-3b-E-M-4b-4c-5a-5b-N-6b]-3′UTR), canonical conservation of essential genes and size (~27.6 kb) typical of IBVs, the Kenyan isolates do not phylogenetically cluster with any genotypes of the 37 IBV lineages and 26 unique variants (UVs). Excluding the spike gene, genome sequences of A374/17 and A376/17 are only 93.1% similar to each other and 86.7–91.4% identical to genomes of other AvCoVs. All five non-spike genes of the two isolates phylogenetically cluster together and distinctly from other IBVs and turkey coronaviruses (TCoVs), including the indigenous African GI-26 viruses, suggesting a common origin of the genome backbone of the Kenyan isolates. However, isolate A376/17 contains a TCoV-like spike (S) protein coding sequence and is most similar to Asian TCoVs (84.5–85.1%) compared to other TCoVs (75.6–78.5%), whereas isolate A374/17 contains an S1 gene sequence most similar to the globally distributed lineage GI-16 (78.4–79.5%) and the Middle Eastern lineage GI-23 (79.8–80.2%) viruses. Unanswered questions include the actual origin of the Kenyan AvCoVs, the potential pathobiological significance of their genetic variations, whether they have indeed established themselves as independent variants and subsequently spread within Kenya and to the neighboring east/central African countries that have porous live poultry trade borders, and whether the live-attenuated Mass-type (lineage GI-1)-based vaccines currently used in Kenya and most of the African countries provide protection against these genetically divergent field variants.

## 1. Introduction

Infectious bronchitis virus (IBV) is an avian gamma-coronavirus (AvCoV; *Coronaviridae* family) that causes upper respiratory tract, enteric, renal, and reproductive disease (infectious bronchitis; IB) in domestic fowl and is associated with severe economic losses in the domestic poultry industry globally [1]. This virus was first reported as the causative agent of “respiratory disease of chicks” in the U.S. in the 1930s; however, it has been detected globally over the decades since and has also been reported in non-domestic Galliforms [2,3,4]. Available data from the U.S.A. and Europe have shown that effective control of clinical IB depends on combinations of several strategies including good biosecurity, accurate diagnosis, proper vaccination, and surveillance [5]. Despite these mitigation efforts, novel IBV variants repeatedly emerge (created by mutations, recombination events, inefficient vaccination and prophylactic regimes, etc.), which contribute to localized IB outbreaks [5,6,7].

The RNA genome of IBV (~27.6 kb in size) contains six genes flanked by 5′- and 3′- untranslated regions (UTRs) [8,9,10]. Gene 1 (Replicase 1ab; Rep1ab) encodes 15 nonstructural viral RNA transcription proteins (nsp2–16), whereas genes 2, 3, 4, and 6 encode the structural spike (S), envelop (E), membrane (M), and nucleocapsid (N) proteins, respectively [11,12]. Additional accessory proteins with wide configurations are encoded by gene 3 (3a and 3b), gene 4 (4b and 4c), gene 5 (5a and 5b), and putative 7 protein (6b). Although their roles in viral infection and pathogenesis are not fully elucidated, some are postulated to modulate growth kinetics and antiviral responses (3a/3b and 5a/5b), whereas others (4b/4c and 6b) are hardly reported in the literature [13,14,15,16]. The IBV genome is generally organized as 5′UTR-[1ab-S-3a-3b-E-M-4b-4c-5a-5b-N-6b]-3′UTR. The S glycoprotein is the largest of the AvCoV proteins, and it defines tissue tropism and mediates binding and fusion of viral and host cellular membranes [17,18]. Its S1 subunit contains hypervariable regions (HVRs) and antigenic sites, and the complete S1 is currently used to classify IBV into six genotypes (GI to GVI) which comprise 32 distinct lineages (27 in genotypes GI-1 to GI-27, and one each in GII to GVI). Proposed GVII and GVIII, and at least 26 unique variants (UVs) that do not fit into any of the defined lineages, have been identified [19,20,21]. Based on sequence and phylogenetic analyses of the complete S1 gene, putative lineages have been proposed for IBVs recently identified in chicken flocks from China (lineages GI-28 [22] and GI-29 [23], which contain pathogenic isolates that are genetically and antigenically distinct from other IBVs and are endemic in China), and from Trinidad and Tobago (lineage GI-30 [24] containing turkey coronavirus (TCoV)-like isolates).

In Africa, IB was initially reported in the Mediterranean basin countries, and although IBV is commonly detected both in asymptomatic poultry and those exhibiting clinical signs consistent with IB [25], its impact on the African continent is undocumented. Genotypes belonging to eight of the 32 IBV lineages have been reported in Africa, but only GI-26 is described as being indigenously African (unique to the continent), and mostly contains strains from North and West Africa [19]. Other African IBV strains, which are of Eurasian origin, belong to lineages GI-1 (associated with sporadic IB outbreaks in several countries), GI-12 (Nigeria), GI-13 (Algeria, Ethiopia, Morocco, South Africa, and Sudan), GI-14 (Cameroon and Nigeria), GI-16 (Côte d’Ivoire and Nigeria), GI-19 (Algeria, Ghana, Nigeria, South Africa, and Zimbabwe), GI-23 (Egypt and Nigeria), and several UVs from Algeria, Ethiopia, Libya, and Tunisia [19,25,26,27,28]. To date, there is no documented evidence of the presence of AvCoVs in Kenya or the East and Central African regions (except in Cameroon in Central Africa [25]).

Here, we report genome analysis of the first AvCoV sequenced from Kenya and the East African region, as inadvertently discovered by untargeted next-generation sequencing of clinical samples collected from indigenous chickens during a 2016–2018 surveillance for vNDVs in domestic and wild avian species in the country [29].

## 2. Materials and Methods

### 2.1. Samples

The clinical samples used in the current study were collected in January 2017 from indigenous chickens traded at a live bird market (LBM) in Kariobangi North, Nairobi, an indoor market where the chickens are kept in wire-mesh cages (an average of 12 birds per cage). In the Kenyan context, indigenous chickens, which are kept under free-range or caged management systems in backyard farms, are defined as “non-descript crosses of Asiatic meat and game types, Mediterranean egg-types and Bantams of various origins” [30,31,32]. The sampled chickens, which originated from backyard rural farms in eastern (Machakos and Kitui counties) and central (Kiambu county) regions of Kenya, did not exhibit any overt clinical signs of respiratory or other diseases at the time of sampling. From each trader’s consignment of chickens, a cloacal (CL) and an oropharyngeal (OP) swab were collected from each of three randomly selected adult chickens per cage and immediately placed in individual 2.0 mL cryogenic vials (Corning Inc., New York, NY, USA) that contained 1.5 mL of Difco™ brain–heart-infusion broth (Thermo Fisher, Waltham, MA, USA) according to standard procedures [27]. Swabs were immediately stored in liquid nitrogen and preserved at −80 °C until shipment to the Southeast Poultry Research Laboratory (SEPRL) of the United States Department of Agriculture, Agricultural Research Service (USDA-ARS) in Athens, GA, USA for analysis. Sampling was conducted in collaboration with the Directorate of Veterinary Services (DVS) and regional Central Veterinary Laboratories (CVLs) in Kenya.

### 2.2. RNA Extraction

Total RNA was extracted from 50 μL of each CL or OP sample (prepared separately for each bird) using the MagMAX™-96 AI/ND Viral RNA Isolation Kit (Thermo Fisher Scientific, Waltham, MA, USA) and eluted in 50 µL of elution buffer. To selectively deplete ribosomal RNAs (rRNAs) from the host (18S, 28S, and mitochondrial) and bacteria (16S/23S), 12 µL of the total RNAs were treated with an in-house RNaseH rRNA depletion protocol and purified using SPRI beads (Agencourt RNAClean XP Kit; Beckman Coulter Life Sciences, Indianapolis, IN, USA) as recently described [33].

### 2.3. Library Construction and Next-Generation Sequencing

First-strand cDNAs were synthesized from 10 µL of the RNaseH-treated RNAs using sequence-independent, single-primer amplification (SISPA) [34] and random K-8N primer with the SuperScript IV reverse transcriptase (Invitrogen, Waltham, MA, USA), followed by second-strand synthesis using a second-strand synthesis module (NEB Inc., Ipswich, MA, USA) and PCR-amplification of the cDNAs using the Phusion^®^ High-Fidelity PCR Kit (NEB Inc., Ipswich, MA, USA). Libraries were prepared from 5 µL of bead-purified (Agencourt AMPure XP Kit) cDNAs using the Nextera ^TM^ DNA Flex kit (Illumina, San Diego, CA, USA) and pooled (4 nM, 10 µL each) based on quantifications using the Qubit™ dsDNA HS Assay kit (Thermo Fisher, Waltham, MA, USA) and TapeStation HS D5000 ScreenTape Assay (Agilent Technologies, Inc., Santa Clara, CA, USA). A control library (5% PhiX library v3) was added to the diluted library pools (10 pM final concentration), followed by paired-end sequencing (2 × 300 bp) using the 600-cycle MiSeq Reagent Kit v3 (Illumina, San Diego, CA, USA). Each MiSeq run consisted of 48 multiplexed libraries.

### 2.4. Genome Sequence Assembly and Characterization

Raw NGS data were preprocessed and analyzed using a nontargeted taxonomic classification and assembly pipeline developed by BASE₂BIO LLC (Oshkosh, WI, USA), as recently described [35]. The pipeline utilized megahit v 1.2.9 for de novo assembly using default parameters [36]. Open reading frames (ORFs; minimum size set at 50 nucleotides from start to stop codons) and gene coding sequence (CDS) annotations were determined using Geneious Prime^®^ v2022.2.0 (www.geneious.com) as previously described [11]. Putative cleavage motifs in the S1/S2 site (R-X-X-R↓S) and S2′ site (R-X-R↓S) were predicted using ProPserver v1.0 [37]. Potential N-linked glycosylation sites were predicted using NetNGlyc-1.0 (www.cbs.dtu.dk/services/NetNGlyc/). The final assembled consensus sequences were assessed for the presence of mutations (amino acid residue substitutions, insertions, and deletions), and detection of putative recombination events was performed using Recombination Detection Program 4 (RDP4; which besides the original RDP4, includes GENECONV, BootScan, MaxChi, Chimaera, SiScan, LARD, and 3Seq algorithms) v4.101, as described previously [11,38,39].

### 2.5. Sanger Sequencing

Sanger sequencing was used to close short gaps in the consensus sequence obtained from one of the samples from this study (see results section). For this, the SuperScript IV One-Step RT-PCR kit (Thermo Fisher Scientific, Waltham, MA, USA) was used to convert and amplify missing regions using a set of primers specifically designed for the gaps. Amplicons were visualized in 1.5% agarose gel (0.5X TBE), followed by purification of excised DNA bands using the Zymoclean Gel DNA Recovery Kit (Zymo Research, Irvine, CA, USA) and Sanger sequencing using the BigDye™ Terminator v 1 Cycle Sequencing Kit performed on a 3730 xl DNA Analyzer (Thermo Fisher Scientific, MA, USA).

### 2.6. Phylogenetic Analysis

For classification, the S1 gene sequences of representative strains of the 32 IBV lineages, the proposed GVII and GVIII, UVs, and turkey coronaviruses (TCoVs) retrieved from GenBank (*n* = 256 sequences; full-length sequences selected based on classification by Valastro et al. [19]), together with those obtained from this study, were aligned using MAFFT v7.490 [40] executed in Geneious Prime and trimmed using trimAl tool v1.3 [41] to minimize the effects of poorly aligned regions. Phylogenetic analyses were performed using the maximum likelihood method in MEGA6 with 1000 bootstrap replicates of the original data; all positions with less than 95% site coverage were eliminated from phylogenetic tree reconstructions [42]. Phylogenetic analyses of the complete genome and all five non-spike genes were performed as described above using representatives of the IBV lineages and TCoVs. Comparative pairwise homology analyses of the genome and CDS of the Kenyan and other IBVs and TCoVs was performed based on sequence and phylogenetic analyses and a heatmap generated using ClustVis [43].

## 3. Results

### 3.1. NGS-Based Nontargeted Virus Discovery

Forty-eight libraries (i.e., one CL and OP swab from each of 24 birds) derived from backyard farms in the eastern (Machakos and Kitui counties; *n* = 15 and 3 samples, respectively) and central (Kiambu county; *n* = 6 samples) regions of Kenya were successfully prepared, sequenced together (multiplexed) on an Illumina MiSeq sequencer, and processed as described above. The NGS pipeline detected IBV RNA in two CL samples, which contained 9148 and 17,811 IBV-specific read pairs. These two samples were obtained from the same trader, who had sourced the chickens from middle-men at an open-air LBMs in Machakos county. None of the other tested samples (including the OP counterparts of the IBV-positive CL samples) contained detectable quantities of IBV-specific RNAs. In addition to IBV, sample A374 contained RNAs of three other viral agents, i.e., virulent Newcastle disease virus (vNDV), avian rotavirus-G (AvRV-G), and pigeon picornavirus B (PiPV-B) and one avian pathogenic bacterium (*Ornithobacterium rhinotracheale*; ORT), whereas sample A376 contained NDV and two avian pathogenic bacteria (ORT and *Riemerella anatipestifer*). We have recently published complete genome sequences of the vNDVs that co-infect with the IBVs [29].

### 3.2. Sequence Assembly

Complete genome sequences were assembled from the IBV-specific reads obtained in samples A374 and A376 with lengths of 27,619 and 27,767 bases (excluding polyA tail) supported by median read-depth coverage of 94× and 186×, respectively (Table 1). However, the A376/17 consensus sequence contained two gaps (i.e., without read coverage) in the Rep1a gene (227 bases; position 4755 to 4981) and the S-gene (180 bases; position 21,211 to 21,390), which were filled successfully using Sanger sequencing (Table 2). The Kenyan isolates are named IBV/ck/KE/1920/A374/2017 and AvCoV/ck/KE/1922/A376/2017 (GenBank accession numbers: OP899612 and OP899613) and are hereafter abbreviated as A374/17 and A376/17, respectively.

### 3.3. Genomic Organization, Sequence, and Phylogenetic Analysis of the Kenyan IBVs

The genomes of both A374/17 and A376/17 contain 13 ORFs organized as 5′UTR-[Rep1a-Rep1ab-S-3a-3b-E-M-4b-4c-5a-5b-N-6b]-3′UTR (Table 3). Six CDS are longer in A376/17, including S (3618 vs. 3495 bases), 6b (228 vs. 198 bases), Rep1ab (19,886 vs. 19,865 bases), and 4c (165 vs. 150 bases), but are characterized by a shorter 3′-UTR (273 vs. 305 bases) compared to A374/17. Seven CDS (3a, 3b, M, 4b, 5a, 5b, and N) have similar lengths in both isolates. Genomic positions and sizes of nsp2–16 mature peptides proteolytically produced from Rep1a/1ab are shown in Supplementary Table S1.

The complete genome sequences of A374 and A376 showed 87.2% nucleotide identity to each other, or 93.1% identity when the S gene is excluded. Comparative pairwise homologies of the CDS across the genomes of the two Kenyan AvCoVs showed that complete S-genes have the lowest identities (49.1% and 48.7% at the nucleotide and deduced amino acid sequences, respectively), whereas the highest identities are in M (96.4% for both nucleotide and amino acid levels) and 4b (96.1% and 98.9% at the nucleotide and amino acid levels, respectively) (Table 3). Regarding the S1 gene, which is used for AvCoV classification, the two Kenyan isolates share low identities at the level of nucleotide (38.8%) and amino acid (21.6%) sequences.

**Figure 1:**
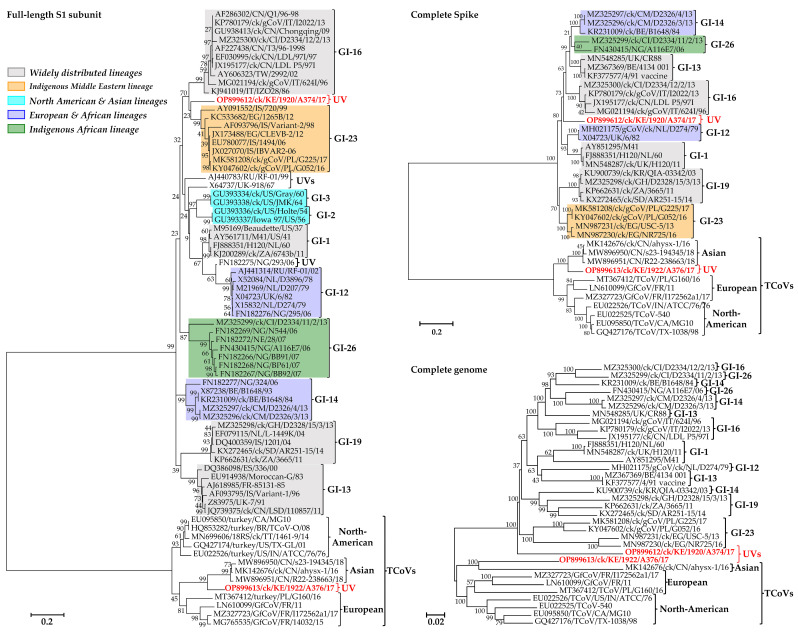
Phylogenetic analyses of the Kenyan AvCoVs (red bold font) and representatives of a selection of lineages (see the detailed tree in Figure S1). Sequence names include GenBank accession numbers, avian species (where applicable), country abbreviation, strain/isolate, and year of sample collection. Lineages are named according to the current IBV classification system (UV indicates unique variant); geographical distribution of the lineages are color-coded [19]. The phylogenetic reconstruction involved 72, 37, and 34 nucleotide sequences (final datasets with 1377, 3233, and 26,625 positions) for the S1, complete S, and complete genome trees, respectively.

Based on the S1 gene, the two Kenyan IBVs identified here do not phylogenetically group with any of the IBVs in lineages GI–GVIII or the UVs [19]; rather, A374/17 clusters non-monophyletically with Eurasian and Middle Eastern viruses (GI-16 and GI-23) and A376/17 with Asian TCoVs (Figure S1). However, the bootstrap value supporting the separation of A374/17 from GI-16 and GI-23 strains is only 23–34% compared to 99% bootstrap support for A376/17 vs. the Asian TCoVs (see Figure 1 and Figure S1). The complete S gene of A374/17 clusters with the globally distributed GI-13 and GI-16 viruses, the West African and European GI-14 and GI-26 viruses, and A376/17 with Asian TCoVs, but the Kenyan isolates branch out from these viruses with bootstrap support of 63% and 100%, respectively; the complete genome sequence tree shows similar clustering with 100% bootstrap support (Figure 1). Phylogenetic analyses based on the other five genes, i.e., gene 1 (Rep1ab), gene 3 (3a, 3b, and envelope), gene 4 (membrane 4a and 4b), gene 5 (5a and 5b), and gene 6 (nucleocapsid and 6b (also referred to as putative 7 protein gene)), differ from the topologies of the S1, complete S, and genome trees in that the two Kenyan isolates cluster together in all five genes (Figure S2).

**Figure 2:**
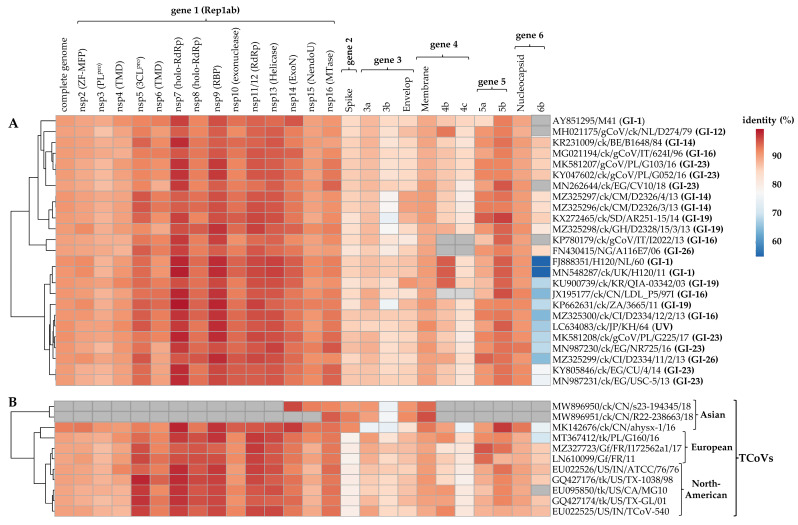
Comparative pairwise homologies of the complete genome sequences and each of the CDS in the six viral genes between the Kenyan AvCoVs A374/17 (panel **A**) and A376/17 (panel **B**) and other strains in genotype I (GI; *n* = 25 strains from 7 lineages) and TCoVs (*n* = 11 strains), respectively. Correlations of the complete genome and respective gene sequences between the strains with reference to the Kenyan isolates are illustrated by the clustering tree on the left side. Colors change from blue to blood orange with increasing nucleotide similarities; gray boxes indicate that the genes in respective strains are not available in public genomic databases. Abbreviations: ExoN—N-terminal exoribonuclease; holo-RdRp—RNA-dependent RNA polymerase holoenzyme; NendoU—nidoviral RNA uridylate-specific endoribonuclease; nsp—non-structural protein; PL ^pro^—papain-like protease; RBP—RNA-binding protein; 3CL^pro^—3C-like protease; TMD—transmembrane domain protein; MTase—(nucleoside-2′-O-)-methyltransferase; ZF-MFP—zinc-finger multifunctional protein.

The complete S protein CDS and S1 gene nucleotide sequences of isolate A374/17 are most similar to viruses belonging to the globally distributed lineage GI-16 (81.8–83.4% and 78.4–79.5%, respectively) and the Middle Eastern lineage GI-23 (82.9–83.1% and 79.8–80.2%, respectively). Conversely, the S protein CDS and S1 gene of isolate A376/17 are most similar to Chinese TCoVs (84.5–85.1% and 81.1–82.2%, respectively) compared to other TCoVs (75.6–75.6% and 72.8–75.5%, respectively). In the non-spike genomic regions, the Kenyan AvCoVs share the highest homologies in five of the 16 nsps in gene 1 (nsp5 (3-C-like protease; 3CL^pro^), nsp7 (RNA-dependent RNA polymerase (RdRp) holoenzyme), nsp9 (RNA-binding protein; RBD), nsp11/12 (RdRp), and nsp13 (helicase)) and the accessory gene 5 (5b), and the lowest homology in 6b (Figure 2).

### 3.4. Analysis of the S Glycoprotein

Because the S gene is critical in IBV’s pathogenesis (binding, entry, and tissue tropism within susceptible host cells and induction of host’s immune responses; [17]), domain analysis of the gene was performed on the two Kenyan viruses compared to strains shown in the complete genome and S-gene trees in Figure 1.

#### 3.4.1. Domain Features of Isolate A374/17

Structural features of isolate A374/17 S glycoprotein are illustrated in Figure S3, among which is the S1/S2 cleavage site with the furin recognition motif **R**-L-**R**-**R**↓**S**, which is located within a 20 amino acid residue region required for cleavage and fusion efficiency [44]. The auxiliary S2′ cleavage site in A374/17 (**P**-S-S-**P**-T-G-**R**↓**S**) has a mutation of the conserved R/K acid residue (consensus motif in IBVs is **P**-X-S-**P**-[**R**/**K**]-X-**R**↓**S**). The membrane fusion peptide of A374/17 has all four residues (**C**TAGPLGFVK**DL**V**C**), which are conserved in CoVs [45], and the number of *N*-linked glycosylation sites (*n* = 23) is well within the range of 19–39 residues expected for CoVs [14].

#### 3.4.2. Mutations in the S1 Subunit Hypervariable Regions (HVRs)

**Figure 3:**
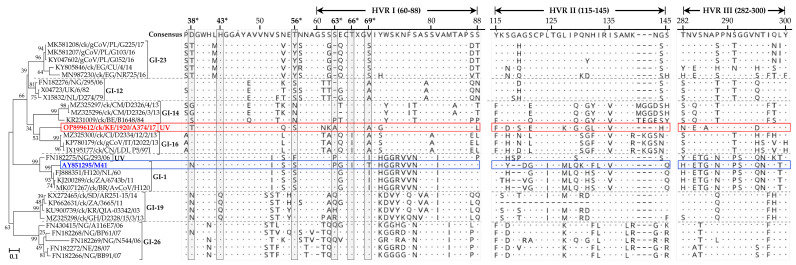
Heterogeneity of the HVRs in the S1 subunit of the Kenyan A374/17 (in red color) compared to other IBVs. Dots and dashes in the alignments indicate identical and missing (or gaps in alignment of) amino acid residues, respectively. Amino acid residue positions are numbered in reference to the consensus sequence. UV indicates a unique variant. The amino acid residues highlighted with asterisks in shaded boxes (“*”; i.e., N38, H43, S56, P63, I66, and T69) are reportedly critical for attachment of the GI-1 prototype strain AY851295/M41 (in blue color) to chicken respiratory tract tissues [48]. The classification is based on Valastro et al. [19].

The three hypervariable regions (HVRs) of IBV’s S1 subunit [46,47] in A374/17 are located at amino acid residues 60–88 (HVRI), 115–142 (HVRII), and 277–295 (HVRIII), all of which are heterogeneous when compared to other IBVs (Figure 3). Notable variations in the HVRI include the six residues described previously as critical for attachment to respiratory tract tissues of the GI-1 prototype M41 strain (residues N38, H43, S56, P63, I66, and T69; GenBank accession number AY851295; [48]), in which A374/17 has mutations at positions N38T (also found in Egyptian and Polish GI-23 strains), P63A (residue deleted in GI-12 and GI-23 strains), I66T (also found in all other analyzed strains except GI-16 strains), and T69A (also found in Eurasian and West African GI-12 and GI-16 strains). Residue H43 is conserved in all analyzed sequences, except in the GI-19 strains from South Africa, Ghana, Sudan, and South Korea. Of the analyzed sequences, S56 is conserved in the Kenyan A374/17 and European and Nigerian GI-12 strains, unlike in Cameroonian and Belgian GI-14 (S56N mutation), Egyptian and Polish GI-23 strains (S56Y mutation), and a Nigerian UV strain (FN182275/NG/293/06; S56F mutation). Several other variations between the Kenyan and other IBVs in the HVRs II and III are clearly observed in Figure 3.

#### 3.4.3. Domain Features of Isolate A376/17

Figure S4 shows the S glycoprotein domain features of isolate A376/17 compared to Eurasian and North American TCoVs. One of the conserved features observed across all the TCoVs used in the analysis is the S1/S2 cleavage site with the consensus motif **R**x**RR**↓**S**, except for the U.S. strains TCoV/IN/ATCC/76 and TCoV/MN/310/96, which have an alanine (A) instead of the conserved serine (S) amino acid residue. Furthermore, all North American TCoVs used in the analysis have the conserved motif (NQGR↓S) in the S2 subunit resembling the auxiliary S2′ cleavage site of IBVs [49], which is also present in some French Guinea fowl CoVs (GfCoVs). Although A376/17 and the Chinese TCoVs have the critical arginine (R) residue at the S2′ site, the conserved asparagine (N) and glycine (G) residues are substituted with serine (S) residues (*S*Q*S*R↓S), whereas TCoVs from France and Poland have proline (P) or S residues (*P*/*S*QGR↓S). The membrane-fusion peptide (FP) is conserved in the Polish and North American TCoVs, and in French GfCoVs (consensus motif **C**IASRGGSFTNLA**DL**T**C**; conserved residues are underlined [45]) compared to substitutions in the Kenyan A376/17 (*n* = 2), Chinese (*n* = 1), and the French (*n* = 5) TCoVs. The antigen-binding region (FabR; 45 amino acid residues located at the S1 C-terminus [49,50]) of the Kenyan A376/17 and the Asian TCoVs are identical (except one amino acid difference; G vs. S) compared to high variations in the European TCoVs and GfCoVs. The numerous amino acid variations in the HVR are expected in TCoVs [51]. The S protein of A376 has 26 *N*-linked glycosylation sites, which is consistent with other CoVs [14].

### 3.5. Analysis of Recombination Events

Because recombination significantly contributes to the continuous emergence of IBV variants, RDP4 was used to investigate possible recombination events in the two Kenyan AvCoVs, and strains from the most widely distributed lineages (GI-1, GI-13, GI-16, and GI-19), North American/Asian lineages (GI-2 and GI-3), European/African lineages (GI-12 and GI-14), European lineage GI-21, indigenous Middle Eastern/African lineages (GI-23 and GI-26, respectively), and TCoVs (see Figure 1). One of the recombination signals was detected in Rep1ab (nsp11/12; RdRp) of both A374/17 and A376/17 with the Canadian strain TCoV/MG10 and Sudanese strain AR251-15 (lineage GI-19) as the major and minor parental strains, respectively (Table 4).

**Figure 4:**
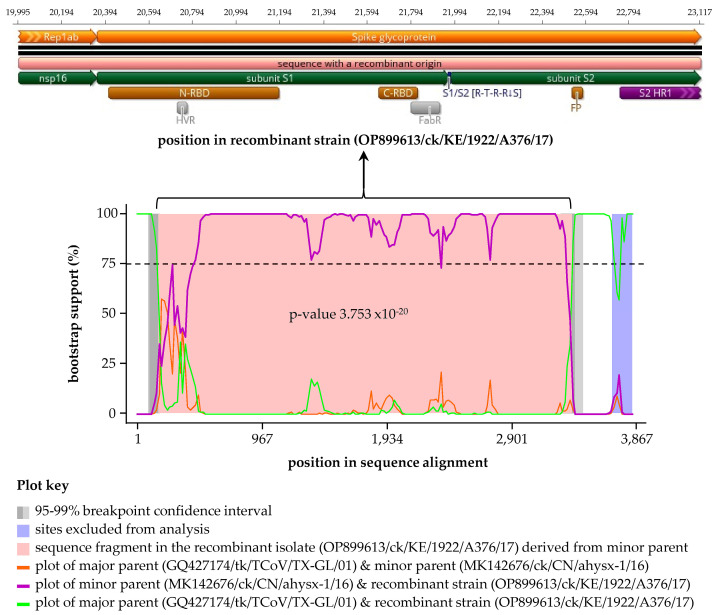
Schematic representation of the recombinant sequence detected using RDP4 in the Kenyan A376/17 strain. The recombination signal was supported by six methods (see Table 4) with a North American major parent (strain TX-1038/98) and an Asian minor parent (strain ahysx−1/16 (see Table 4 footnotes)). The top panel illustrates the sequence in the recombinant Kenyan isolate A376/17 transferred by recombination event, which corresponds to the pink area flanked by recombination breakpoints on the left and right boundaries (genomic positions 19,995 and 23,117, respectively) in the recombination signal plot in the bottom panel. Also shown are structural/domain features in the recombinant sequence, which include N-/C-terminal receptor-binding domains (N-/C-RBD), hypervariable region (HVR), antigen-binding region (FabR), S1/S2 cleavage site, fusion peptide (FP), and heptad repeat region 1 (HR1); see Figure S4 for detailed features of A376/17. Definitions: “minor parent” represents the strain (i.e., ahysx-1/16) closest to the sequence fragment transferred to the recombinant strain (i.e., Kenyan isolate A376/17) via recombination event; “major parent” represents the strain (i.e., TX-1038/98) closest to the sequence surrounding recombination breakpoints in the recombinant isolate.

A recombination event was detected by six of the nine RDP4 methods [38] in the Kenyan isolate A376/17 with the Asian ahysx-1/16 and North American TX-1038/98 strains predicted as the minor and major parental sequences, respectively (Table 4 and Figure 4). Note that here, “minor parent” represents the strain (i.e., ahysx-1/16) closest to the sequence fragment that has potentially been transferred to the recombinant isolate (i.e., Kenyan isolate A376/17) via a recombination event from the “major parental” strain (i.e., TX-1038/98) closest to the sequence surrounding recombination breakpoints in the recombinant isolate. This recombinant signal was not detected in any other of the strains used in the analyses. It is of note that the Chinese TCoV strain ahysx-1/16 isolate was itself reported as being of recombinant origin between an IBV backbone and a TCoV-like S gene donor [52], but our analysis did not give evidence that strain ahysx-1/16 and the Kenyan isolates share the same parents. Nonetheless, the S sequence of A376/17 is more similar to ahysx-1/16 than to any other published sequence, and the recombinant fragment itself (3123 nucleotides in length) covers 39.5% of the C-terminus of nsp16, the full-length S1 gene, and approximately 75%% of the S2 region.

Four and six other recombination signals were detected in A374/17 and A376/17, respectively, but these were considered possible misidentifications of recombination because of one or more of the following reasons: (1) one or both breakpoints could not be identified; (2) the recombinant signal represented only trace evidence of a recombination event, i.e., the *p*-value was less than 10^−5^ cutoff and/or was supported by fewer than five out of the nine RDP4 detection methods [38]; and (3) if there was more than 30% probability that one or both the major and minor parental strains are likely to be the actual recombinant. For instance, a recombination event signal was predicted (four methods; *p*-value of 4.6 × 10^−4^) in Rep1ab (nsp14–15) of A376 with the Kenyan A374 and a GI-13 4/91-vaccine strain (GenBank accession number KF377577) as the major and minor parents with 93.9% and 86.9% identities, respectively, but A374 was suggested to be the actual recombinant.

## 4. Discussion

Molecular and serological surveys in the backyard and commercial poultry flocks have reported IBV variants in the Mediterranean basin and Western and Northern African countries [27,53], but epidemiological and genomic data on IB and its impact on the poultry industry in Africa are scarce. This is despite IBV being one of the most common and important viral agents associated with respiratory disease and severe reduction in egg production in chickens, with IB outbreaks occurring in commercial and backyard flocks regardless of their vaccination status [54]. Contributing factors to this situation include a lack of surveillance initiatives for poultry viruses and inadequate genomic sequence data of detected isolates, most of which remain uncharacterized. The situation worsens in Central and Eastern Africa, where only Cameroon (in Central Africa) out of the 16 countries in these regions reported five GI-14, GI-16, and GI-19 strains during a 2013 surveillance of commercial poultry [25]. Towards the southernmost African region with 11 countries, only South Africa, Zimbabwe, and Botswana have reported serological detections of anti-IBV antibodies and a few GI-1, GI-13, and GI-19 variants [53,55,56,57,58].

Here, we report for the first time in Kenya and the Eastern African region, two AvCoV variants identified using random untargeted NGS of samples collected from rural backyard indigenous chickens during a 2016–2018 surveillance for vNDVs in domestic and wild avian species in the country. The IBV RNAs were detectable in the CL samples and not in their counterpart OP samples, which suggests the possibility that the Kenyan isolates A374/17 and A376/17 are gastroenteric, but this remains to be further investigated because birds in general shed viruses for longer durations via the cloacal route compared to the respiratory routes. This is not unexpected, because the gastrointestinal tract is capable of supporting IBV replication [59]. Furthermore, the absence of overt signs of respiratory or other diseases in the chickens at the sampling time agrees with gastrointestinal tract IBV infections that are yet to be demonstrated to result in enteropathogenesis [60]. We have recently reported sequences of novel subgenotype V.3 vNDVs in A374 CL and A376 OP samples [29], and five low pathogenic influenza A subtype H9N2 viruses [61] from other samples from the same LBM as those in the current study (collected at the same time and originating from the same Machakos county). It is highly likely that these respiratory viruses have been disseminated to other Kenyan and East African regions, because the LBMs and associated trade routes form the backbone of the poultry industry in these regions [29,61].

Although the genome organization (5′UTR-[Rep1a-Rep1ab-S-3a-3b-E-M-4b-4c-5a-5b-N-6b]-3′UTR) and size (~27.6 kb) of the two Kenyan isolates are as expected of AvCoVs with canonically conserved essential genes [12,62], they are phylogenetically distinct from other established IBVs in lineages GI–GVIII and UVs [19]. The S gene sequences place the Kenyan isolate A374/17 in a large cluster containing the globally distributed GI-16 and the Middle-Eastern GI-23 viruses, but based on the S1 gene, this isolate is closer to GI-23 than GI-16 viruses. Other African AvCoVs phylogenetically group with viruses in five of the most widely distributed lineages GI-1 (e.g., South African strain 6743b/11), GI-13 (e.g., Moroccan-G/83), GI-14 (e.g., Nigerian and Cameroon strains 324/06 and D2326/13, respectively), and GI-19 (e.g., Sudanese and South African strains AR251-15/14 and 3665/11, respectively). Conversely, isolate A376/17 is closer to Asian (and French) TCoVs than it is to the North American TCoVs, but it is also a UV distinct from all analyzed TCoVs (95–100% bootstrap support). Although the other five viral genes do not strictly group the IBVs in accordance with their respective S1-based lineage classifications, the two Kenyan isolates cluster together distinctly from other IBVs, UVs, and TCoVs with bootstrap support values of 97% (gene 1), 46% (gene 3), 50% (gene 4), 54% (gene 5), 98% gene 6, and 48% (gene 6b; putative 7 protein). The uniqueness of the Kenyan isolates is corroborated by numerous amino acid variations (mutations) observed in S glycoproteins (e.g., HVRs, S1/S2 and S2′ cleavage motifs, FP, and FabR) when compared with previously reported IBVs and TCoVs.

The current IBV classification is predicated on the S1 gene being the most variable and immunogenic (epitomic) genomic region, and the most commonly sequenced [19], but the question arises whether one gene alone sufficiently reflects the actual variability of AvCoVs. The inconsistencies in the topologies of the full-length S1 gene, complete S CDS, and complete genome sequence trees vs. the trees of the other five viral genes observed in the current study reiterates the challenge in the classification and nomenclature of new AvCoVs that emerge continuously because of their high propensity for recombination. It is, therefore, not far-fetched to conclude that the evolution and epidemiology of these viruses could potentially be more reliably tracked by expanding genomic studies from using the S1 gene to using complete genome sequences, full-length of the S protein CDS, or by combining data from phylogenies of each gene.

The higher nucleotide identity among the two Kenyan isolates in the non-spike genomic regions (93.1%) and the phylogenetic grouping together of all five viral genes except the S-gene suggest that their genomic backbones have a common origin. These Kenyan isolates are closest to but group distinctly from the Middle Eastern IBVs (isolate A374/17) and Asian TCoVs (isolate A376/17) and are distantly related to the indigenously African GI-26 viruses (as described by Valastro et al., [19]), suggesting that they are indigenous (unique) to Kenya. It is common to refer to IBVs as indigenous to specific geographical regions where they were originally isolated, e.g., the North American Massachusetts (lineage GI-1) and Arkansas (lineage GI-9), the Dutch D274 and British UK/6/82 (lineage GI-12), and the Chinese QX (GI-19) viruses [19,63]. Whereas some variants remain restricted to circulating only within avian subpopulations in the geographical regions of their original discovery, other variants can persist long enough to adaptively evolve into independent strains that consequently spread and establish themselves in entirely different geographical areas where they can gain substantial economic importance in the poultry industry, even becoming responsible for disease outbreaks [19,64]. The questions as to what extent the Kenyan variants are established in eastern Kenya and whether they may have spread within the country or east Africa, remain to be determined. The possibility of any of these scenarios is of concern in view of the highly unregulated live poultry trade networks in the country and the region [65].

Together, data from the current study raise questions about the actual origin and evolution of the Kenyan AvCoVs, whether they are also circulating in commercial poultry flocks, and whether migratory wild birds and other non-domestic avian species frequently found scavenging together with rural backyard poultry play roles in the evolution and dissemination of the variants. From our sequence and phylogenetic analyses, the two Kenyan isolates are more similar to each other than to any other sequenced AvCoV sequences over the majority of the genomic backbone, suggesting a shared evolutionary history, and lack of similarities to other sequenced full-length AvCoVs. The S-protein coding sequence of isolate A376 appears to be of recombinant origin similar to but distinct from other sequenced TCoVs such as the Asian and North American TCoVs (at 85.9% and 79% nucleotide similarity, respectively), and only distantly related to other IBVs. This is a known phenomenon, in which the majority of TCoVs originate from a recombination of an IBV backbone with an S-gene fragment of often unknown origin [18,52,66]. The ability of AvCoVs to micro-evolve into novel variants via recombination of S1 sequence fragments from different backgrounds is consequential because they could confer on the variants the ability to infect new host tissues or naïve susceptible hosts (diversification of tissue tropism and host range), for instance, if the changes enhance the susceptibility of the S gene to proteolytic activation or increase affinity to receptor binding and efficiency to gain cellular entry (heightening of pathogenicity) [18,67]. Novel variants could also gain the ability for immune escape and unresponsiveness to vaccinations.

In most African countries, live-attenuated and inactivated Mass-type-based vaccines are the main control method [68]. Kenya has two official government-approved Mass-type vaccines, i.e., CEVAC^®^ BI L (strain B-48; used via eye-drop or spray method) and CEVAC^®^ Corymune 7K (strain M-41; used subcutaneously). However, even with the availability of these vaccines, vaccination rates are low outside commercial poultry farming, which is an issue of concern because ~80% of the Kenyan poultry are under non-commercial settings (i.e., free-range birds kept in small-scale flocks of ~30 birds). Some commercial farmers also tend to consult private veterinarians for vaccine options other than the government-approved ones. Based on the phylogenetic and sequence analyses presented in this study, the two Kenyan UVs presented in this study are clearly genetically different from the GI-1-based vaccine strains, which are currently used in Kenya and other African countries. A lack of evidence as to whether the Kenyan UVs cause clinical disease makes it necessary to review the protection of the Mass-type vaccines used in Kenya, because there is evidence that variations of as little as 5% in the S1 gene sequence can negatively affect vaccine efficacy [12].

## 5. Conclusions

We have reported for the first time in Kenya and the Eastern Africa region the presence of AvCoVs in backyard chickens. Our analyses have demonstrated that the two Kenyan AvCoVs are UVs that are distinct from each other but likely share a common origin not yet represented in the literature, and they do not group with any of the genotypes or strains belonging to the established lineages or other UVs. It is yet to be elucidated whether the genomic variations of the two Kenyan AvCoVs when compared to other previously reported strains are of any pathobiological significance. Outstanding questions include the actual origin of the Kenyan AvCoVs; whether they have indeed established themselves as independent variants in the eastern county of Machakos; and, if so, to what extent they may have spread to the rest of the country and the neighboring east African countries that have porous borders in terms of trade of live poultry. Nevertheless, data from this study underscore the need for active surveillance, identification, and characterization of the variants that are evolving and circulating in the country and the region, because effective vaccination against novel variants requires tailor-made vaccines.

## Figures and Tables

**Table 1:** Summary of NGS data obtained from two cloacal (CL) samples from Kariobangi North LBM which contained IBV RNA. Other microbial agents detected in the CL samples are indicated.

Isolate	NGS Read Pairs			Median Coverage Depth (Reads)	Consensus Sequence Length (Bases)	Other Agents Detected *	
	Total	Host-Specific	IBV-Specific			Viral	Bacterial
A374/17	844,763	15%	9148	94×	27,619	vNDV; PiPV-B; AvRV-G	ORT
A376/17	328,958	3.5%	17,811	186×	27,767	NDV	ORT; *R. anatipestifer*

* Abbreviations: AvRV-G—avian rotavirus group G; vNDV—virulent Newcastle disease virus; ORT—*Ornithobacterium rhinotracheale*; PiPV-B—pigeon picornavirus type B; *R. anatipestifer*; *Riemerella anatipestifer.*

**Table 2:** Primers used to close two gaps in sequence A376/17 using Sanger sequencing.

Target Genomic Region: Position (Gap Length)	Primer Name	Primer Sequence (5′ to 3′)	Annealing Tm (°C)	Amplicon Size (Genomic Region)
Rep1a (nsp3) gene: 4755–4981 (227 bases)	IBV1F	TGA TGT GGA CTA CAC GAA CG	62.7	635 bases (4539–5173)
	IBV1R	GTG TCA ATG GCA ACT TGG AGT C		
Spike gene: 21,211–21,390 (180 bases)	IBV2F	AGG CTG GTA ATG TGG TAG	59.0	701 bases (20,993–21,693)
	IBV2R	TAT AGT GCC AAC GCC TCT G		

**Table 3:** Genomic organization and comparative homologies of isolates A374/17 and A376/17 identified in the current study. The position and nucleotide (nt) and amino acid (aa) lengths of the untranslated regions (5′-/3′-UTRs) and protein-coding sequences (CDS) of the genes are indicated.

Isolate	Feature	5’-UTR	Gene 1		Gene 2		Gene 3			Gene 4			Gene 5		Gene 6		3’-UTR
			Rep1a	Rep1ab	S1	S2	3a	3b	E	M	4b	4c	5a	5b	N	6b	
A374/17	position	1–515	516–12,347	516–20,380	20,331–21,947	21,948–23,825	23,825–23,998	23,998–24,177	24,170–24,490	24,468–25,139	24,140–24,424	25,345–25,494	25,491–25,688	25,685–25,933	25,876–27,105	27,114–27,311	27,312–27,616
	length (nt)	515	11,832	19,865	1617	1878	174	180	321	672	285	150	198	249	1230	198	305
	length (aa)	-	3943	6621	539	625	57	59	106	223	94	49	65	82	409	66	-
A376/17	position	1–516	517–12,369	517–20,402	20,353–21,972	21,973–23,970	23,970–24,143	24,143–24,322	24,315–24,638	24,616–25,287	25,288–25,572	25,493–25657	25,641–25,838	25,835–26,083	26,026–27,255	27,263–27,490	27,491–27,763
	length (nt)	516	11,853	19,886	1620	1998	174	180	324	672	285	165	198	249	1230	228	273
	length (aa)	-	3950	6628	540	665	57	59	107	223	94	54	65	82	409	75	-
similarity to each other	nt	96.7%	92.7%	93.0%	40.8%	56.3%	93.7%	94.4%	93.8%	96.4%	96.1%	94.7%	93.4%	93.6%	91.3%	93.4%	98.7%
	aa	-	96.8%	97.6%	33.1%	63.0%	93.0%	98.3%	96.2%	96.4%	98.9%	83.3%	95.4%	92.7%	96.8%	76.9%	-

**Table 4:** Recombination events detected in the Kenyan isolates A374/17 and A376/17. The analysis was performed using RDP4 and involved 42 complete genome sequences from different IBV lineages (described in the text). Nucleotide identities between the transferred fragment in the recombinant and the major/minor parent sequences are indicated.

Sequence	Breakpoint			“Major Parent” Sequence (nt) ^a^	“Minor Parent” Sequence (nt) ^b^	Recombination Event Confirmation	
	Start	End	Gene			Detection Algorithm	*p*-Value
A374/17	12,346	13,046	nsp11/12 (RdRp)	EU095850/TCoV/ CA/MG10 (89.4%)	KX272465/SD/ AR251-15 (95.4%)	RDP, GENECONV, BootScan, MaxChi, SiScan, Chimaera, 3Seq	8.71 × 10^−6^
A376/17	12,337	13,037	nsp11/12 (RdRp)	EU095850/TCoV/ CA/MG10 (89.5%)	KX272465/SD/ AR251-15 (99.1%)	RDP, GENECONV, BootScan, MaxChi, SiScan, Chimaera, 3Seq	2.42 × 10^−37^
	19,955	23,117	nsp16/spike	GU213200/TX-1038/98 (93.8%)	MK142676/CN/ahysx-1/16 (85.9%)	GENECONV, BootScan, MaxChi, Chimaera, SiScan, 3Seq	3.75 × 10^−20^

^a^ “major parent” indicates a sequence in another strain most closely related to the sequence surrounding the fragment transferred to the recombinant strain through recombination. ^b^ “minor parent” indicates the sequence closely related to the fragment in the recombinant strain.

## Data Availability

The nucleotide sequence data of the Kenyan IBV isolates reported in this paper have been submitted to GenBank database and have been assigned the accession numbers OP899612 and OP899613.

## Supplementary Materials

The following supporting information can be downloaded at: https://www.mdpi.com/article/10.3390/v15020264/s1, Table S1. Positions and sizes (nucleotides) of nsp2-nsp16 mature polypeptides proteolytically produced from Rep1a (nsp2-nsp11) and Rep1b (nsp12-nsp16) of the Kenyan AvCoVs reported in the current study. Abbreviations: DUF—protein domain of unknown function; ExoN—N-terminal exoribonuclease; Hel—helicase; holo-RdRp—RNA-dependent RNA polymerase (RdRp) holoenzyme; NendoU—nidoviral RNA uridylate-specific endoribonuclease; nsp—non-structural protein; PL pro—papain-like protease; RBP—RNA-binding protein; Rep—Replicase; 3CL pro—3C-like protease; TMD—transmembrane domain protein; 2′-O-MTase—(nucleoside-2′-O-)-methyltransferase; and ZF-MFP—zinc-finger multifunctional protein. Figure S1: Phylogenetic clustering of the two Kenyan IBVs identified in the current study (bold font) and representatives of other AvCoVs based on the nucleotide sequences of the full-length S1 gene. The lineages are named based on the current IBV classification system and are color-coded based on their geographical distribution [19,20,21,22,23,24]. The phylogenetic reconstruction involved 256 sequences, with the final dataset having 1433 positions. Sequence names include GenBank accession numbers, avian species (where applicable), country, strain/isolate, and year of sample collection. Figure S2: Phylogenetic analyses of Kenyan AvCoVs (in red font) and other strains based on genes 1, 3, 4, 5, 6, and 6b. IBV lineages and TCoVs are highlighted in bold (in brackets) and blue text, respectively. Figure S3: Schematic representation of structural features of the Kenyan isolate A374/17 S glycoprotein compared to representatives of other IBV lineages (selected based on phylogenetics in Figure 1 and Figure S1). Dots and dashes indicate identical and missing (or gaps in alignment of) amino acid residues, respectively. Amino acid residues that are conserved across IBVs are underlined in the consensus sequence. Features of subunit S1 include N-terminal signal peptide (SP); N- and C-terminal domains (S1-NTD/CTD, which harbor the receptor-binding domains (RBD); and hypervariable regions (HVR I-III). Shown is a 20-amino acid residues S1/S2 cleavage site (running from positions P14 to P6′), which consists of a core region (8-residues; position P6 to P2′with **R**-X-[**K**/**R**]-**R**↓**S** canonical motif; “X” is any amino acid residue; and “↓” indicates cleavage position illustrated by a red dotted vertical line) flanked by two solvent accessible regions (8-residue; P7 to P14, and a 4-residue; P6 to P2′). Note the backward and forward numbering of P1-P14 and P1′-P6′, respectively, starting at the conserved R immediately upstream of the cleavage S1/S2 site. Subunit S2 domains include auxiliary S2′ cleavage motif (**P**-X-S-**P**-**R**-X-**R**↓**S**) [49], fusion peptide (FP with consensus motif **C**IASRGGSFTNLA**DL**T**C**), heptad repeat regions (HR1 and HR2), and transmembrane/non-cytoplasmic domains (TM/Non-CTD). Purple vertical bars represent predicted N-linked glycosylation sites (*n* = 23 in A374/17; positions of the asparagine (N) residues in N-X-S/T sequons are indicated; X is any amino acid residues). Figure S4: Schematic representation of structural features of the Kenyan isolate A376/17 S glycoprotein compared to Eurasian and North American TCoVs. Dots and dashes indicate identical and missing (or gaps in alignment of) amino acid residues, respectively. Conserved amino acid residues are underlined in the consensus sequence. Features of subunit S1 include N-terminal signal peptide (SP), N-and C-terminal receptor-binding domains (S1-N/C-RBD), a hypervariable region (HVR), and an antigen-binding region (FabR; *n* = 45 amino acid residues in length). The boxed amino acid residues in the FabR are reported to be critical for antibody neutralization in the North American TCoVs [50]. The S1/S2 cleavage site (**R**-X-**R**-**R**↓**S**; “X” is any amino acid residue and “↓” is the cleavage position illustrated by a red dotted vertical line) is shown. Subunit S2 features include an auxiliary S2′ cleavage motif with NQGR↓S consensus observed in the North American and some French TCoVs [49]), fusion peptide (FP; consensus motif **C**IASRGGSFTNLA**DL**T**C**), heptad repeat regions (HR1 and HR2), and transmembrane/non-cytoplasmic domains (TM/Non-CTD). Purple vertical bars represent predicted N-linked glycosylation sites (*n* = 26 in A376/17; positions of the asparagine (N) residues in N-X-S/T sequons are indicated; X is any amino acid residues).
