# Supplementary material for: Unique Variants of Avian Coronaviruses from Indigenous Chickens in Kenya
Source: Viruses. 2023 Jan 17;15(2):264. doi: 10.3390/v15020264 (PMC9961390; doi:10.3390/v15020264)
Supplement: Supplementary file 1 [file viruses-15-00264-s001.zip › viruses-2143282-supplementary-Figure S1.pdf]

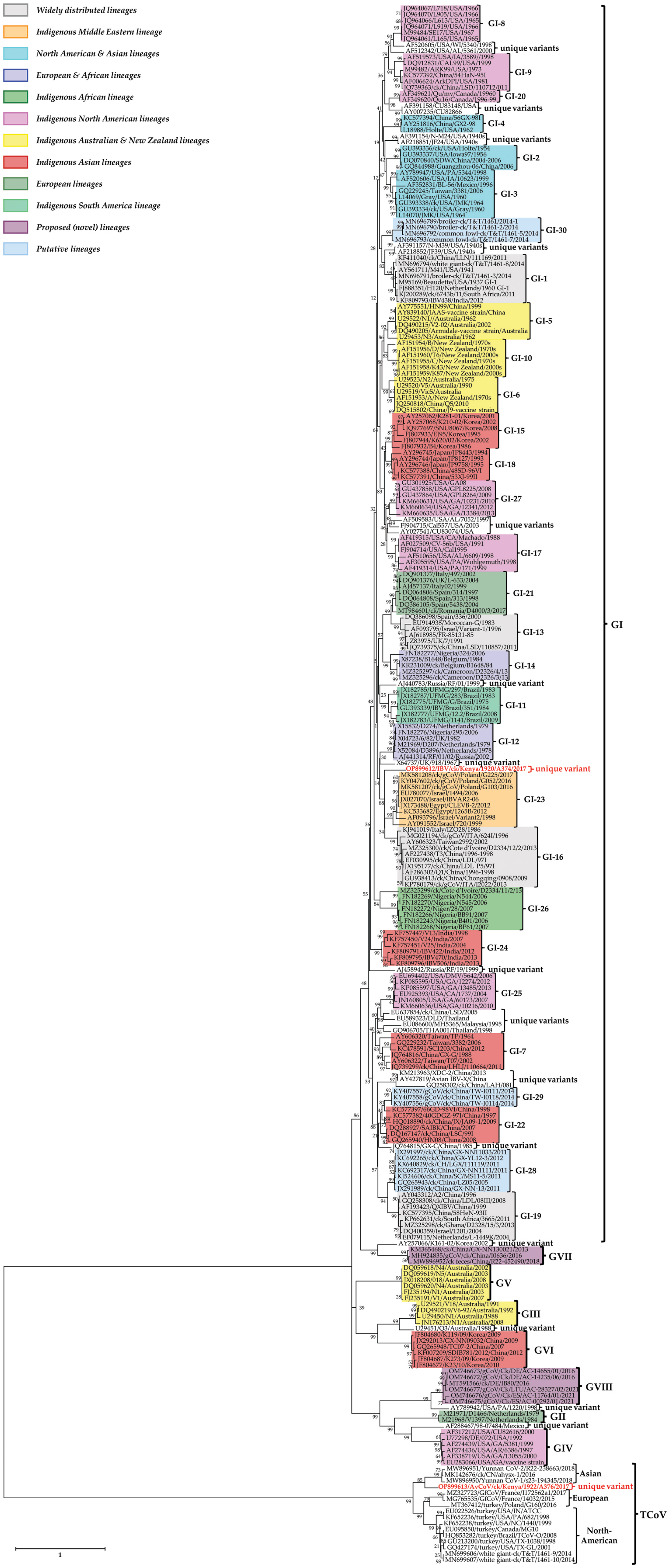

Widely distributed lineages
Indigenous Middle Eastern lineage
North American & Asian lineages
European & African lineages
Indigenous African lineage
Indigenous North American lineages
Indigenous Australian & New Zealand lineages
Indigenous Asian lineages
European lineages
Indigenous South America lineage
Proposed (novel) lineages
Putative lineages
GI-8
unique variants
GI-9
GI-20
GI-4
GI-2
GI-3
GI-30
GI-1
GI-5
GI-10
GI-6
GI-15
GI-18
GI-27
GI-17
GI-21
GI-13
GI-14
unique variant
GI-11
GI-12
OP899612/IBV/ck/Kenya/1920/A374/2017
GI-23
GI-16
GI-26
GI-24
GI-25
GI-7
GI-29
GI-22
GI-28
GI-19
GI
GVII
GV
GIII
GVI
GVIII
GII
GIV
Asian
OP899613/AvCoV/ck/Kenya/1922/A376/2017
European
North-American
TCoV
1
