# Supplementary figures and images for: Unique Variants of Avian Coronaviruses from Indigenous Chickens in Kenya
Source: Viruses. 2023 Jan 17;15(2):264. doi: 10.3390/v15020264 (PMC9961390; doi:10.3390/v15020264)

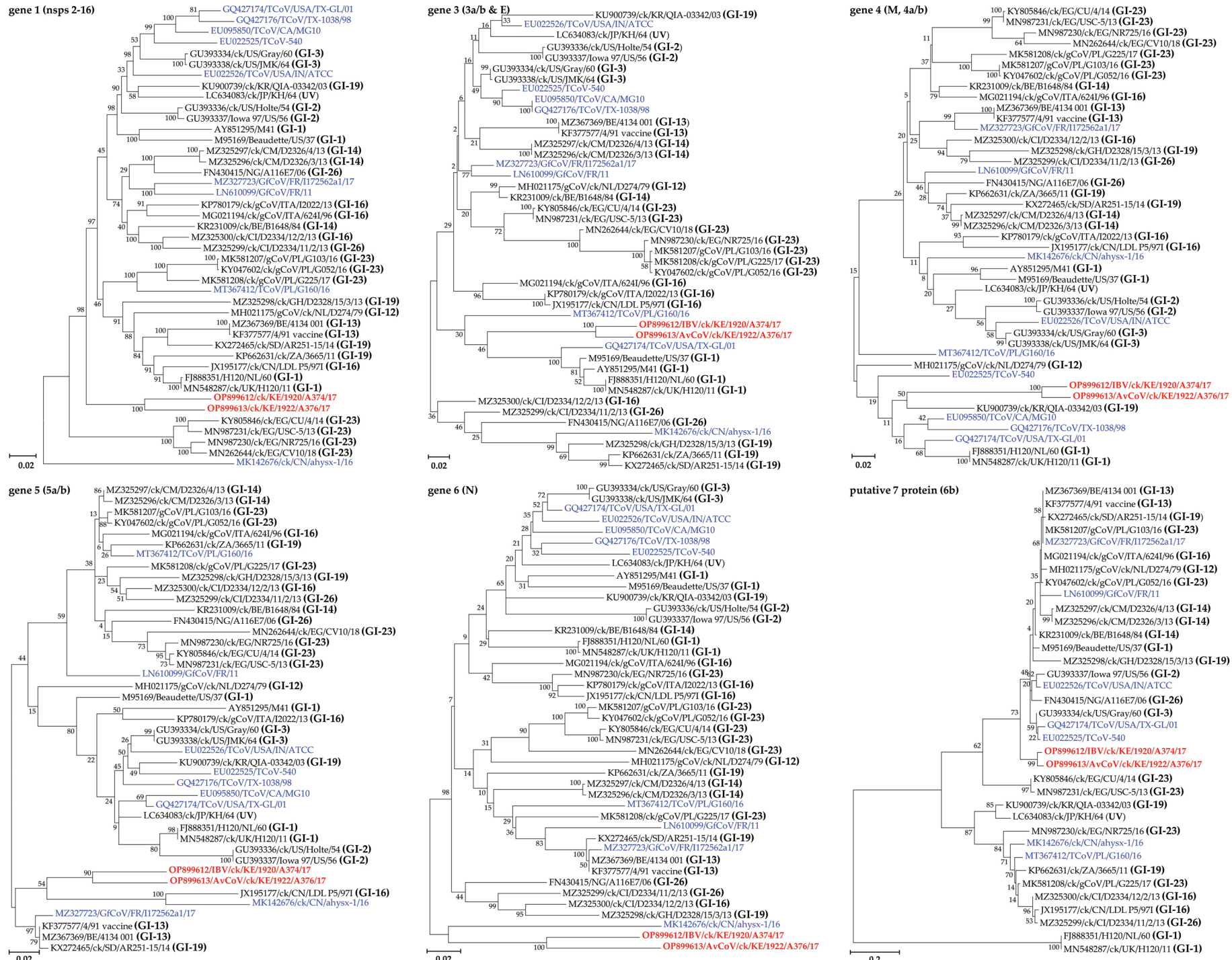

Supplement: Supplementary file 1 [file viruses-15-00264-s001.zip › viruses-2143282-supplementary-Figure S2.pdf]

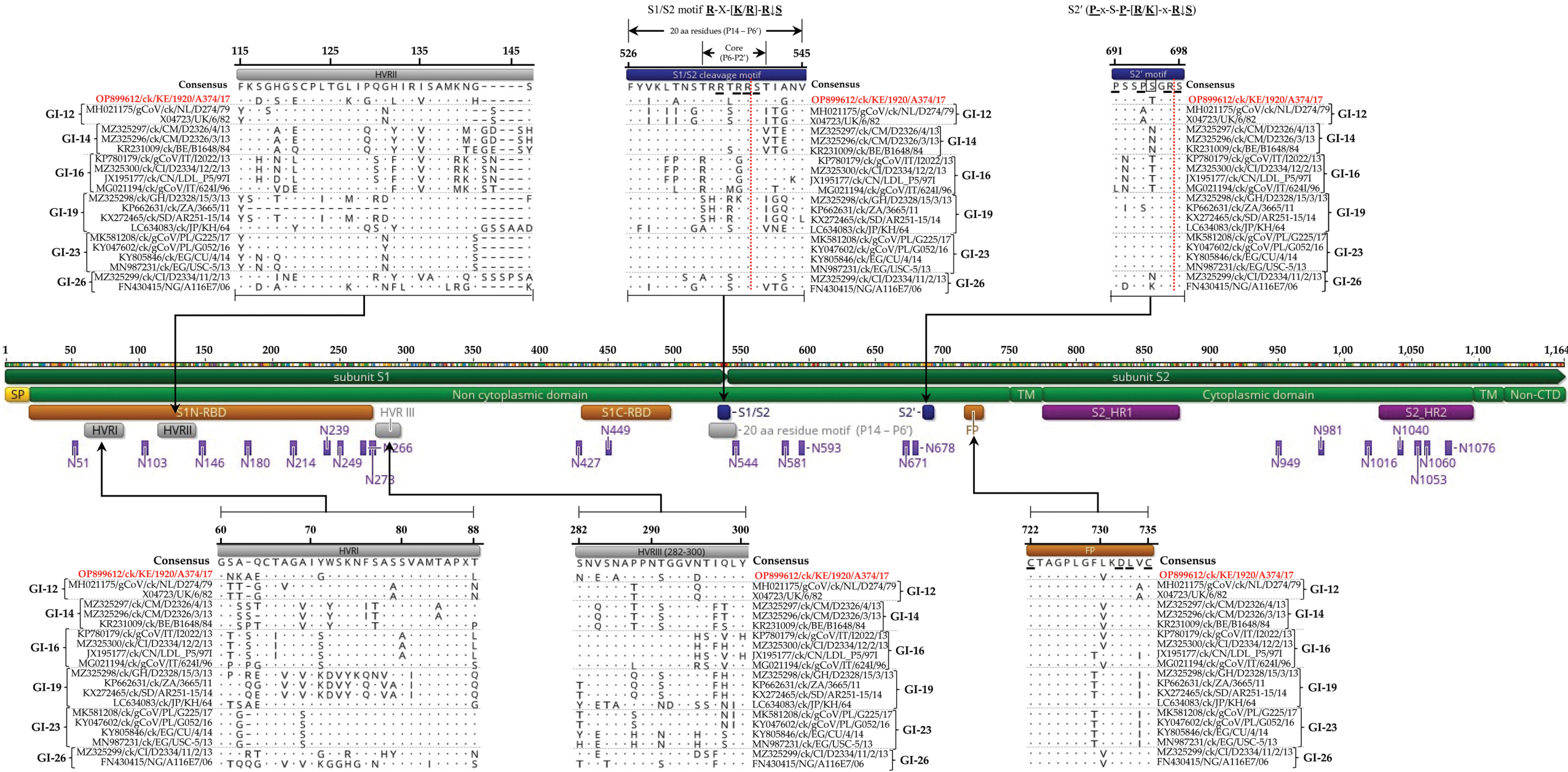

Supplement: Supplementary file 1 [file viruses-15-00264-s001.zip › viruses-2143282-supplementary-Figure S3.pdf]

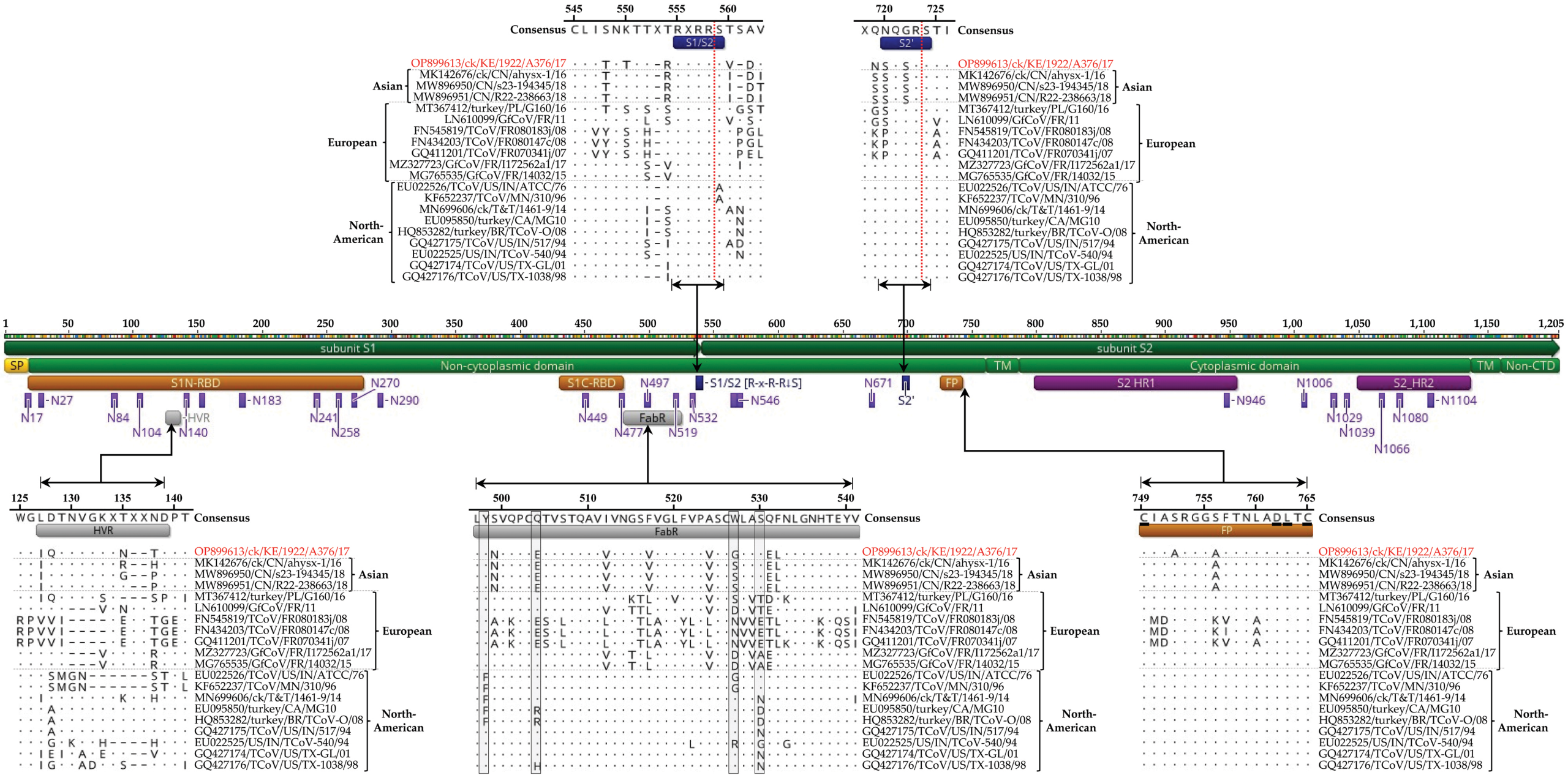

Supplement: Supplementary file 1 [file viruses-15-00264-s001.zip › viruses-2143282-supplementary-Figure S4.pdf]

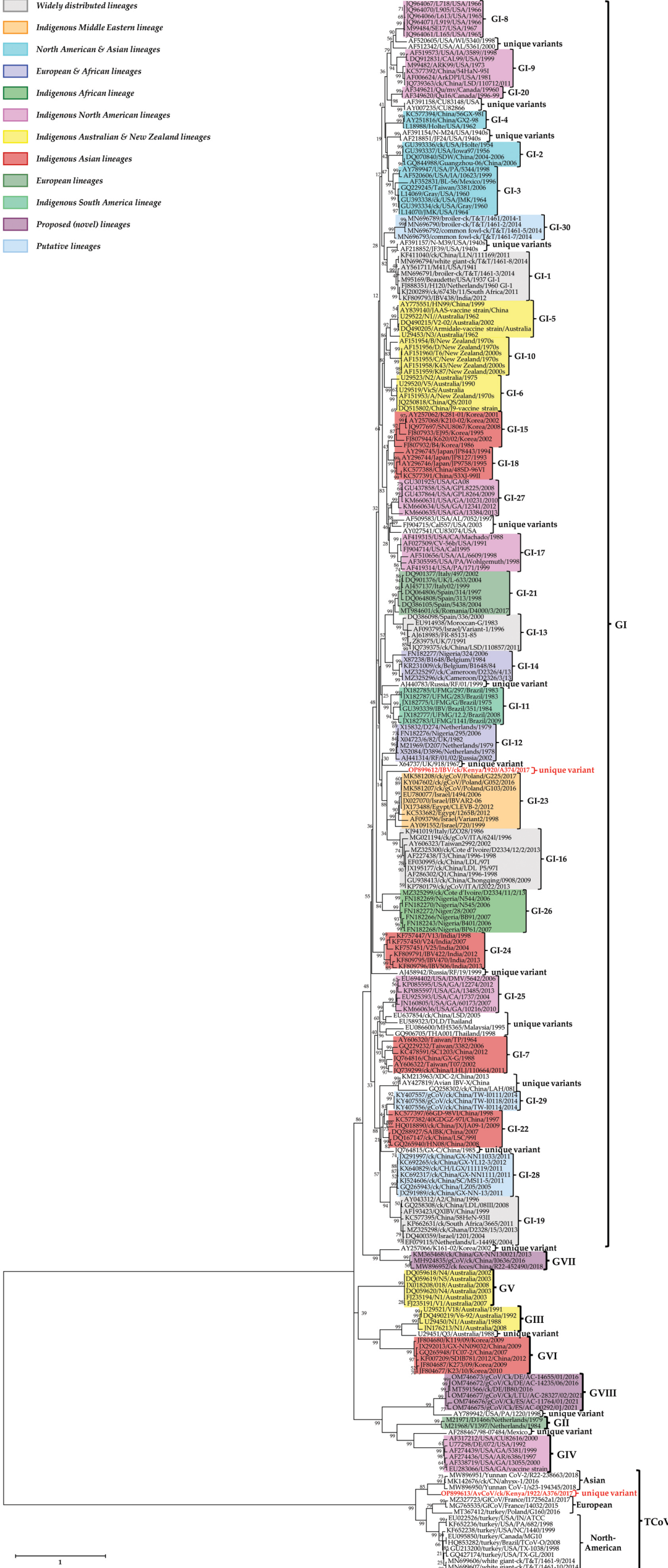

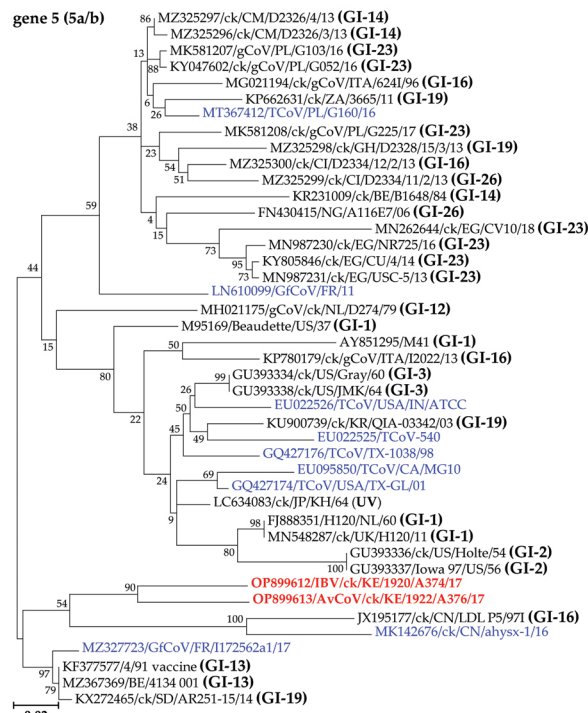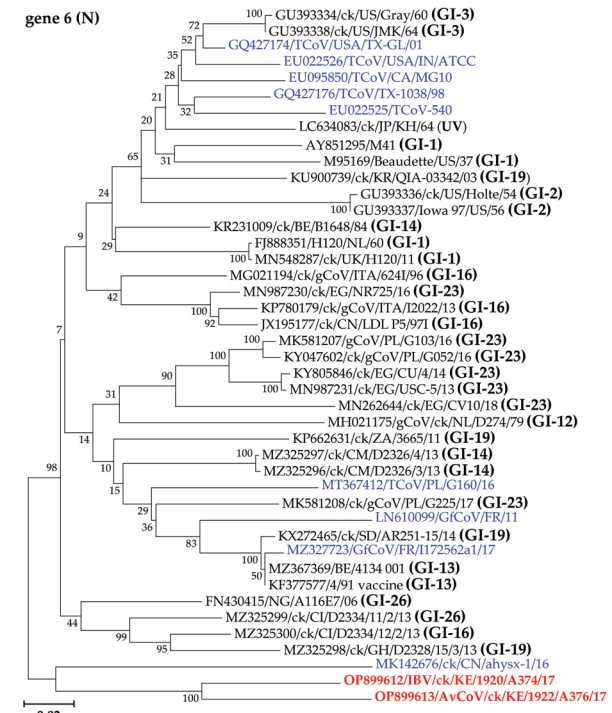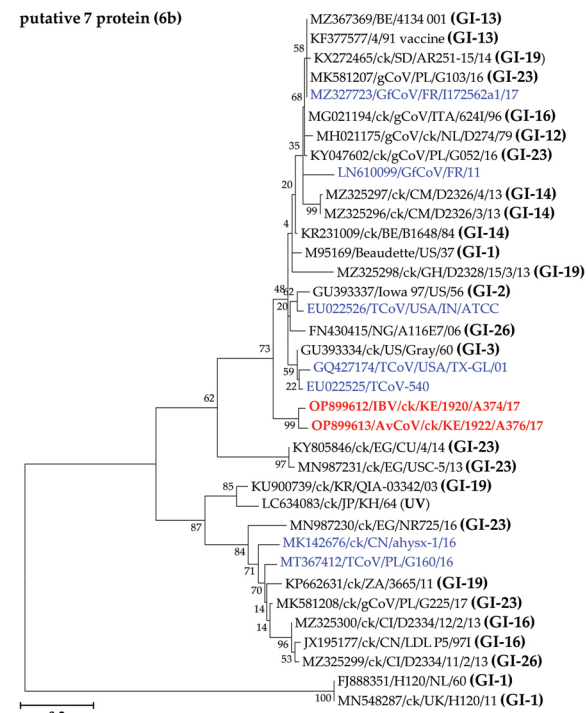

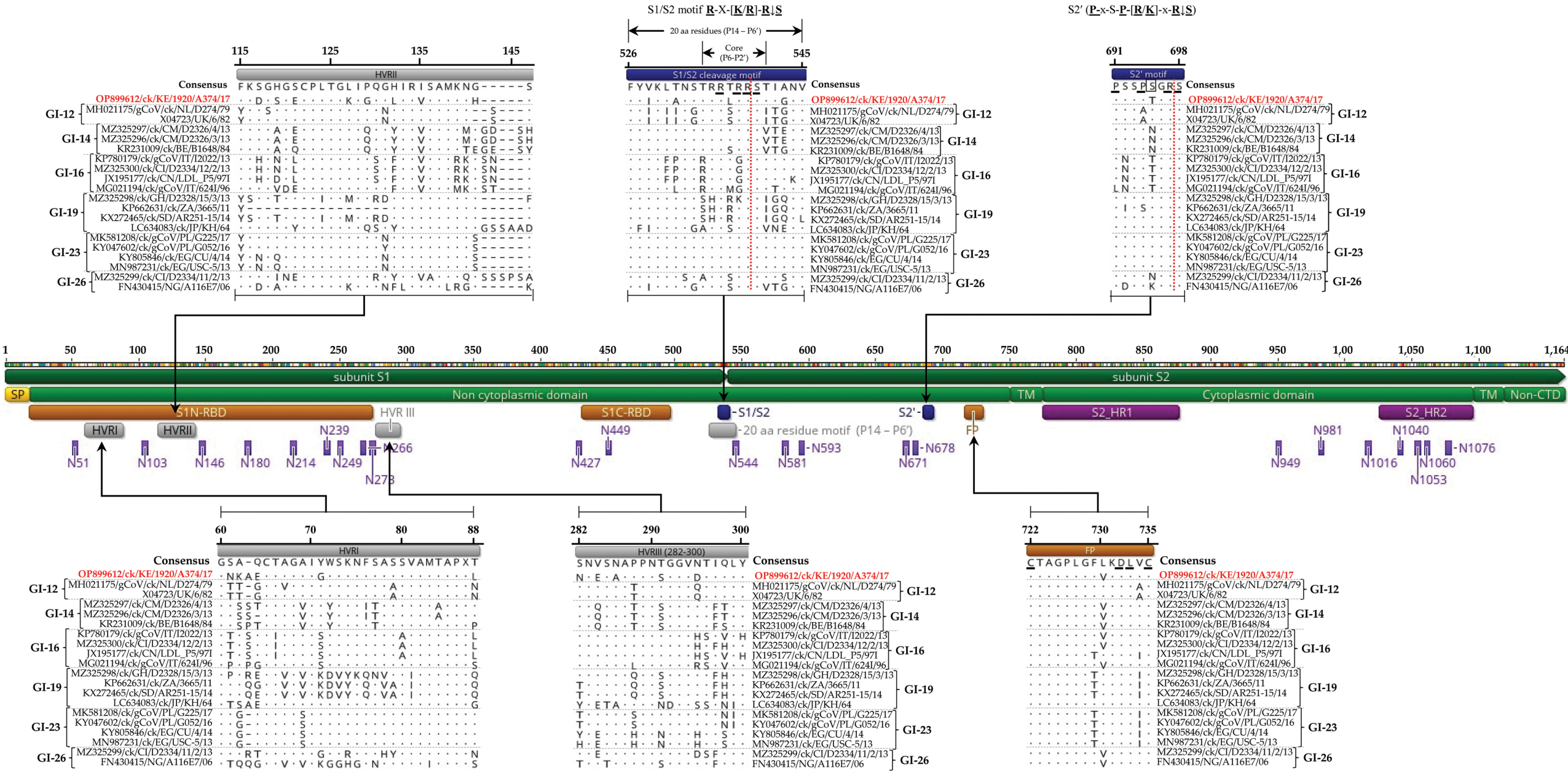

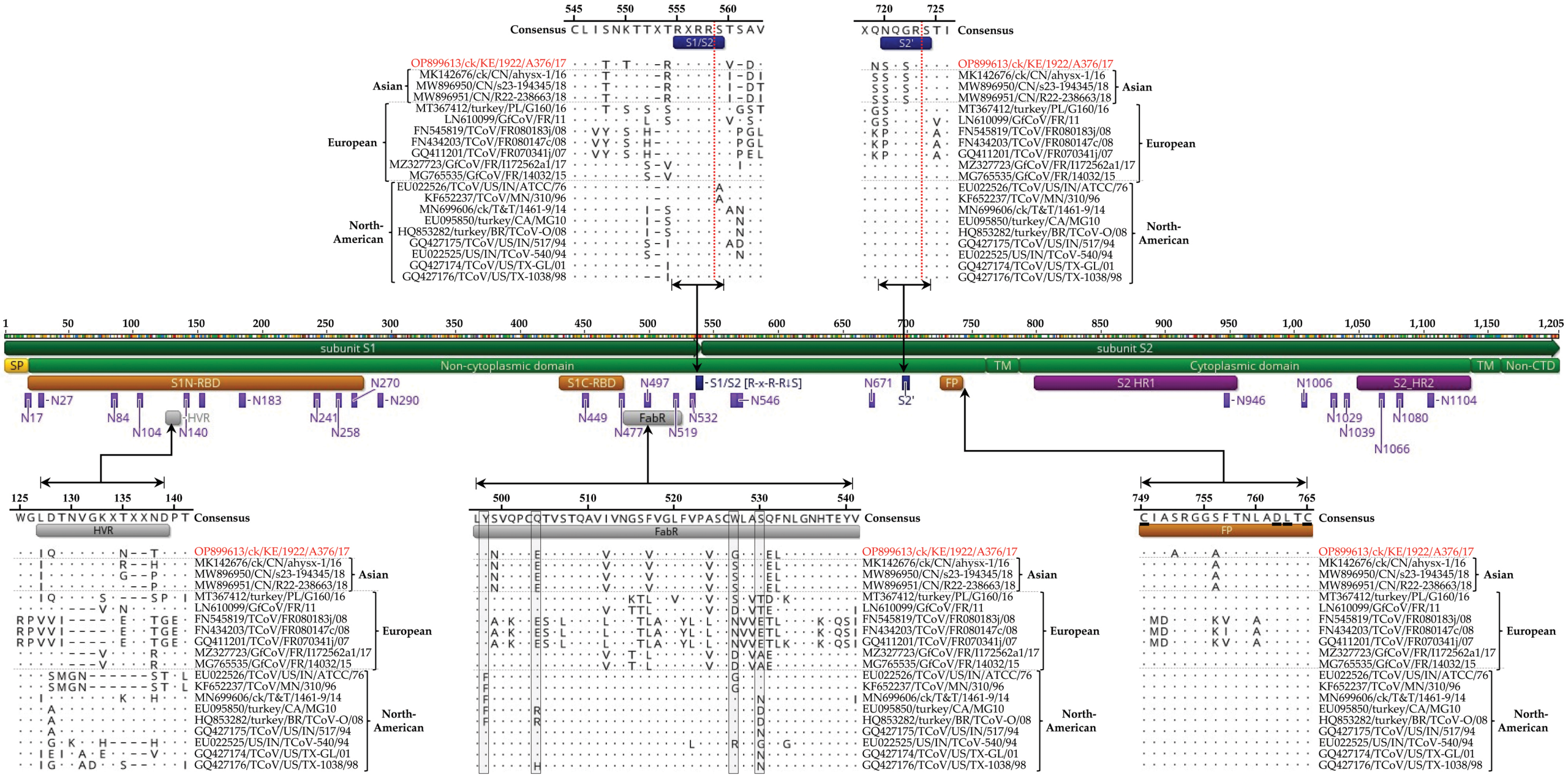

Supplement: Supplementary file 1 [file viruses-15-00264-s001.zip › viruses-2143282-supplementary-figures.pdf]
